# Exercise and high-fat feeding remodel transcript-metabolite interactive networks in mouse skeletal muscle

**DOI:** 10.1038/s41598-017-14081-w

**Published:** 2017-10-18

**Authors:** Joaquín Pérez-Schindler, Aditi Kanhere, Lindsay Edwards, J. William Allwood, Warwick B. Dunn, Simon Schenk, Andrew Philp

**Affiliations:** 1 MRC-ARUK Centre for Musculoskeletal Ageing Research, University of Birmingham, Birmingham, B152TT UK; 2 School of Sport, Exercise and Rehabilitation Sciences, University of Birmingham, Birmingham, B152TT UK; 3 School of Biosciences, University of Birmingham, Birmingham, B152TT UK; 4 Respiratory Therapy Area Unit, GlaxoSmithKline Medicines Research Centre, Stevenage, SG1 2NY UK; 5 Phenome Centre Birmingham, University of Birmingham, Birmingham, B152TT UK; 6 Department of Orthopaedic Surgery, University of California San Diego, La Jolla, CA 92093-0863 USA; 7 Biomedical Sciences Graduate Program, University of California San Diego, La Jolla, CA 92093-0863 USA; 8 Present Address: Biozentrum, University of Basel, Basel, 4056 Switzerland; 9 Present Address: Environmental and Biochemical Sciences, The James Hutton Institute, Dundee, DD2 5DA Scotland

## Abstract

Enhanced coverage and sensitivity of next-generation ‘omic’ platforms has allowed the characterization of gene, metabolite and protein responses in highly metabolic tissues, such as, skeletal muscle. A limitation, however, is the capability to determine interaction between dynamic biological networks. To address this limitation, we applied Weighted Analyte Correlation Network Analysis (WACNA) to RNA-seq and metabolomic datasets to identify correlated subnetworks of transcripts and metabolites in response to a high-fat diet (HFD)-induced obesity and/or exercise. HFD altered skeletal muscle lipid profiles and up-regulated genes involved in lipid catabolism, while decreasing 241 exercise-responsive genes related to skeletal muscle plasticity. WACNA identified the interplay between transcript and metabolite subnetworks linked to lipid metabolism, inflammation and glycerophospholipid metabolism that were associated with IL6, AMPK and PPAR signal pathways. Collectively, this novel experimental approach provides an integrative resource to study transcriptional and metabolic networks in skeletal muscle in the context of health and disease.

## Introduction

The prevalence of metabolic diseases and associated pathologies is rapidly rising, representing the main cause of death worldwide and substantial economical burden^1–3^. The development of metabolic diseases is linked with disruption of multiple interconnected ‘omic’ layers (e.g. transcriptome, epigenome and metabolome) through different genetic factors and environmental cues^4,5^. Among the metabolically active tissues affected by metabolic diseases, skeletal muscle represents a central modulator of whole body metabolic health and a key therapeutic target via lifestyle interventions such as exercise and dietary modification^6^. Both obesity and type 2 diabetes (T2D) disrupt the skeletal muscle transcriptome and metabolome compare to healthy individuals, which is mainly characterised by alterations in glucose, lipid and amino acid metabolism^7–10^. For instance, several human studies have reported a consistent down-regulation of genes associated with oxidative metabolism in skeletal muscle from T2D patients^7^. Moreover, at the metabolic level, obese subjects have lower content of several amino acids in skeletal muscle, while short-chain acylcarnitine species are higher in blood^8^. Therefore, decoding the mechanisms regulating different skeletal muscle ‘omic’ layers in response to exercise and diet represent an attractive strategy to identify novel therapeutic targets for metabolic diseases.

Although informative, the separate analysis of ‘omic’ datasets does not allow comprehensive elucidation of the integrative physiology underlying metabolic diseases. This limitation has begun to be addressed via the development of trans-omic (i.e. combining multiple ‘omic’ layers) approaches^4,5^. For instance, Williams and colleagues recently analysed several ‘omic’ datasets from the BXD mouse genetic reference population, which through correlation analysis between datasets (e.g. transcriptomics, proteomics and phenome) discovered several factors regulating metabolic networks linked with metabolic diseases both at the whole body level and in liver^11^. Similarly, transcriptome and proteome datasets from differentiated human skeletal myocytes have been used to reconstruct genome-scale metabolic models (GEMs), which together with meta-analysis of human studies and reporter metabolite analysis revealed networks linked with mitochondrial pathways were dysregulated in T2D^7^. Lee *et al*. have further expanded this approach by integrating GEMs with transcriptional and protein-protein interaction networks^12^. This allowed the identification of metabolic networks and reporter metabolites dysregulated liver, adipose tissue and skeletal muscle from obese subjects, of which liver mannose metabolism appears to play an important function in the development of insulin resistance in humans^12^. Integration of ‘omic’ datasets has also been used to uncover several mutation linked to T2D that are specifically located within human skeletal muscle enhancers^10^. However, the potential cross talk between the skeletal muscle transcriptome and metabolome in response to diet and exercise remains poorly understood. Moreover, integrated network analysis mainly relies on prediction of regulatory metabolites, thus limiting the accurate integration of the skeletal muscle metabolome with other ‘omic’ datasets.

The principal aim of this study was to assess and integrate transcriptional and metabolic networks regulated by exercise and high fat diet (HFD)-induced obesity in mouse skeletal muscle. We specifically focused on the effects of acute maximal exercise, which in contrast to long-term exercise training, induces a specific gene expression pattern linked with the initial phase of the adaptive response to exercise^13,14^. To discover candidate genes and metabolites, we measured the skeletal muscle transcriptome, metabolome and adapted Weighted Gene Co-expression Network Analysis (WGCNA), a robust method for analysing gene transcription networks^15^. WGCNA has successfully been used to define gene co-expression in several tissues (e.g. liver and skeletal muscle) in metabolic diseases^16,17^, while here we further expanded this approach to integrate transcriptomic and metabolomic datasets in an approach that we refer to as Weighted Analyte Correlation Network Analysis (WACNA).

## Results

### HFD is linked with a gene expression pattern indicative of enhanced lipid catabolism

**Figure 1:**
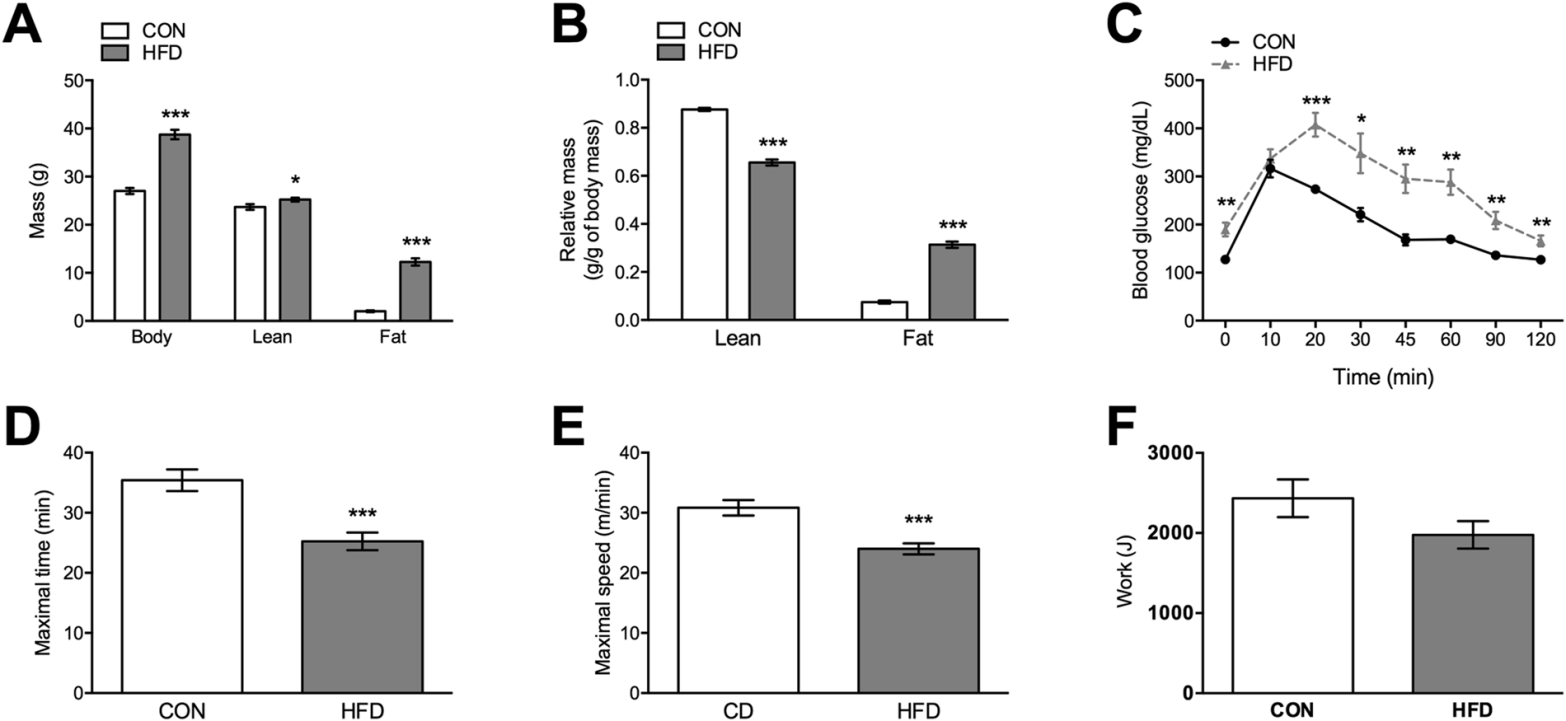
HFD impairs whole body metabolism and exercise performance. Changes in (**A** and **B**) body composition, (**C**) glucose tolerance, (**D** and **E**) exercise performance and (**F**) work were assessed in mice fed either control CON diet or HFD for a period of 10 weeks. Values are mean ± SEM, n = 8–12 mice per group. *p < 0.05, **p < 0.01, ***p < 0.001.

To assess the effects of HFD-induced obesity on skeletal muscle transcript and metabolite responses, we used mice fed either a control (CON) or HFD for 10 weeks that resulted in a significant increase in body weight and fat mass, whereas relative lean mass was reduced by 25% (Fig. 1A,B). In line with the detrimental effects of HFD, both glucose tolerance (Fig. 1C) and exercise performance (Fig. 1D,E) were impaired in HFD mice. However, despite time being different, the work during the maximal exercise test was the same between groups (Fig. 1F). Moreover, since mice ran to exhaustion, relative intensity and, thus, exercise stimulus was comparable between groups.

**Figure 2:**
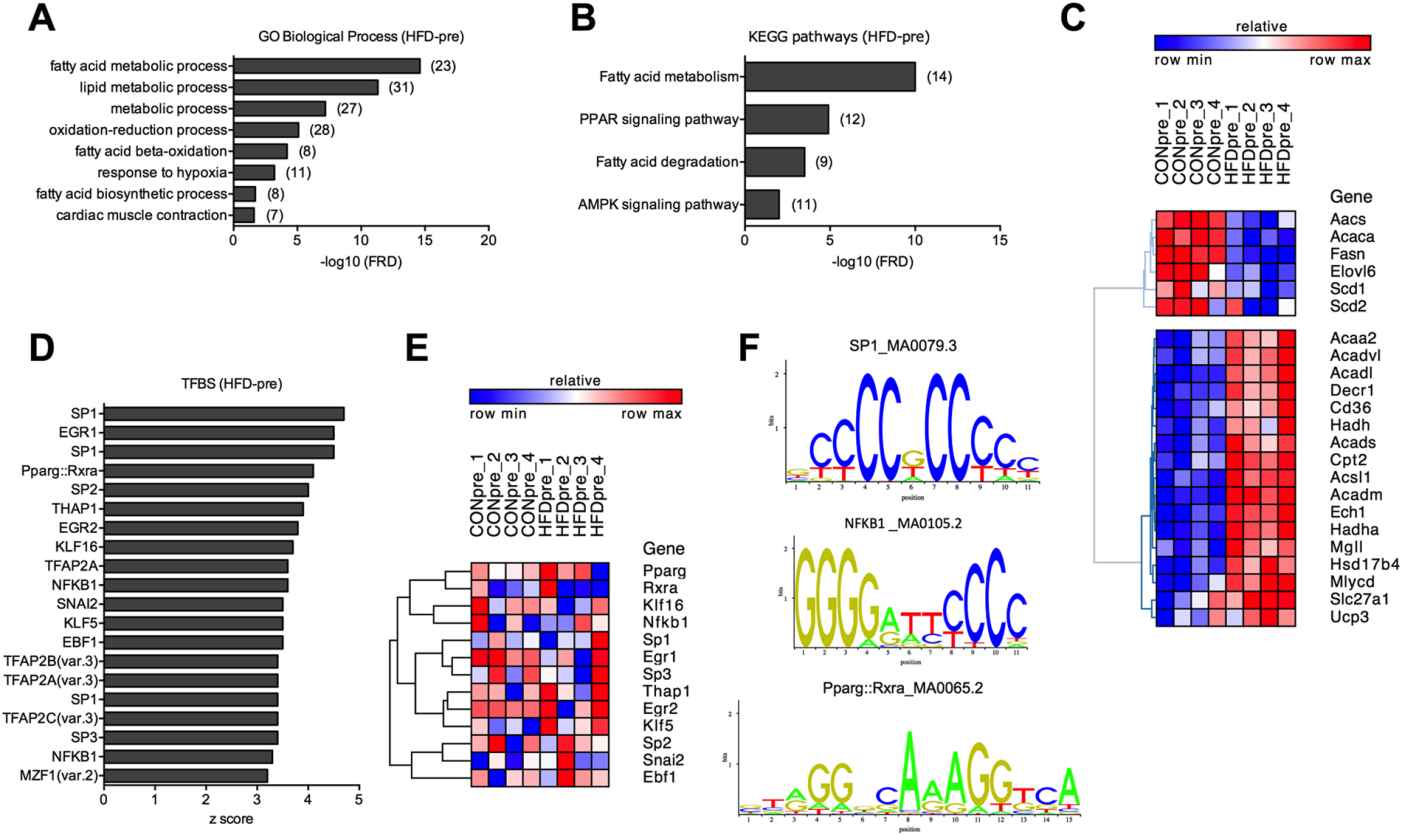
Effects of HFD on the skeletal muscle transcriptome. (**A**) GO biological processes and (**B**) KEGG pathways enriched in HFD-pre DEG (number of genes is shown in brackets). (**C**) Heat map showing cluster analysis of DEG contained in the GO term *fatty acid metabolic process* enriched in HFD-pre. (**D**) Top 20 TFBS enriched in HFD-pre DEG. (**E**) Transcript levels of TFs associated with the top 20 TFBS. (**F**) Representative TFBS logos. n = 4 muscles per group.

At the transcriptional level, HFD induced the up- and down-regulation of 119 and 93 genes in mouse skeletal muscle, respectively (Supplementary Table S1). Gene Ontology (GO) analysis of differentially expressed genes (DEG) in HFD mice in the pre-exercise state (HFD-pre) revealed a strong enrichment of biological processes such as *lipid metabolism*, *response to hypoxia* and *muscle contractility* (Fig. 2A). Moreover, besides fatty acid metabolism, we found peroxisome proliferator activated receptor (PPAR) and AMP-activate protein kinase (AMPK) signalling pathways to be significantly enriched in HFD-pre (Fig. 2B). Both GO and pathway analysis showed an increased expression of several PPAR target genes encoding proteins involved in lipid uptake and catabolism, whereas lipid biosynthesis decreased (Fig. 2C and Supplementary Fig. S1A). Within the AMPK pathway, we observed a down- and up-regulation of 6-phosphofructo-2-kinase/fructose-2,6-biphosphatase 3 (Pfkfb3, −1.8 fold) and TBC1 domain family member 1 (Tbc1d1, 1.4 fold), respectively (Supplementary Fig. S1B), thus consistent with the detrimental effects of HFD on glucose metabolism.

We next performed transcription factor (TF) binding site (TFBS) analysis to predict the subset of potential TFs driving the effects of HFD-induced obesity on the skeletal muscle transcriptome. We identified 46 TFBS substantially (p < 0.01) enriched in HFD-pre skeletal muscle DEG (Supplementary Table S2). Among the top 20 most significant TFBS (Fig. 2D), none of the related TFs were differentially expressed (Fig. 2E), while some TFs were not detected in skeletal muscle. Although the majority of the top 20 TFBS exhibited a motif rich in G and/or C, we found a distinct PPARγ-retinoid X receptor α (RXRα) and nuclear factor κ B (NFκB) TFBS (Fig. 1F), both TFs previously reported to contribute to skeletal muscle biology and energy metabolism^18,19^.

### HFD alters skeletal muscle transcriptome remodelling induced by acute maximal exercise

**Figure 3:**
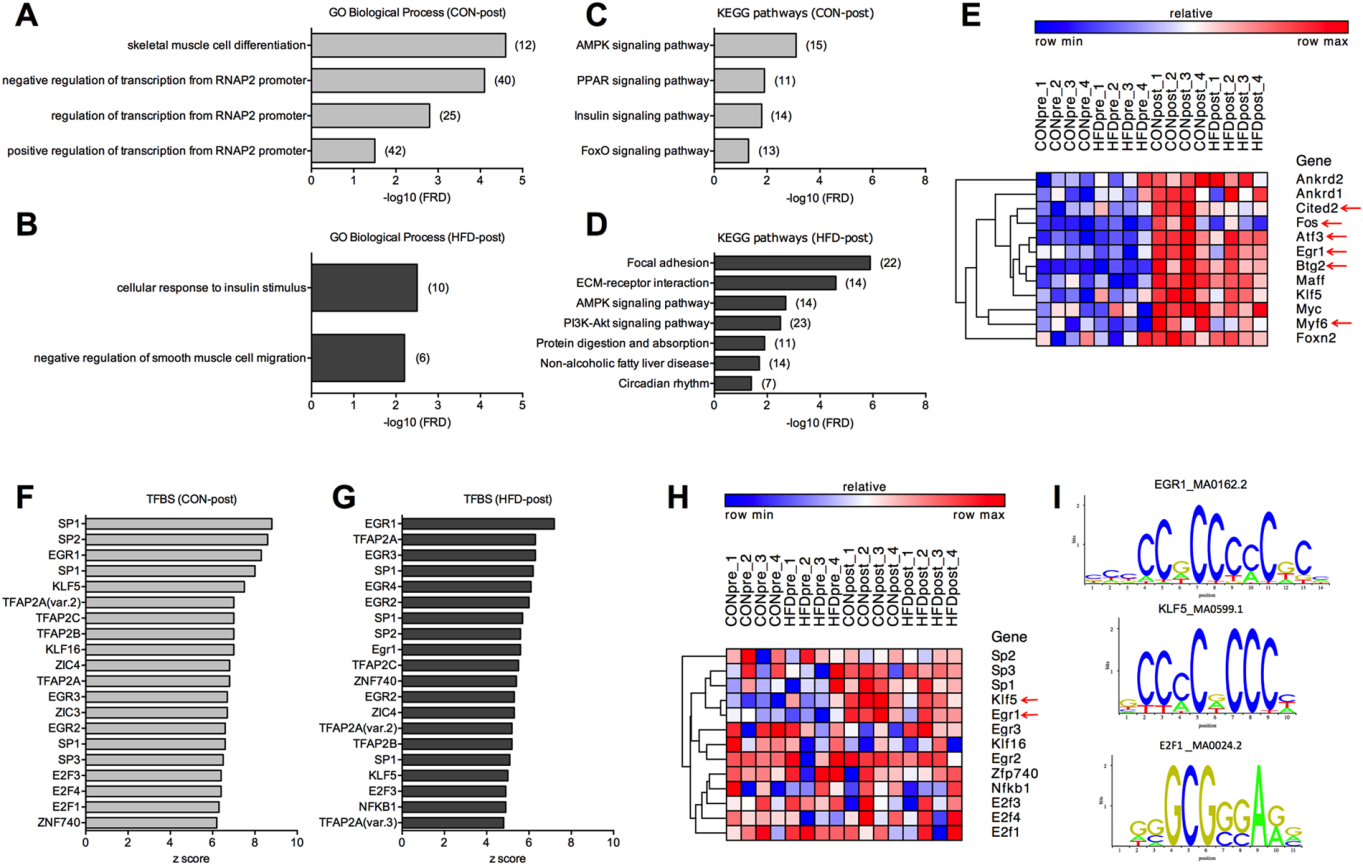
Transcriptional response to acute maximal exercise in CON and HFD mice. (**A** and **B**) GO biological processes and (**C** and **D**) KEGG pathways enriched in CON-post and HFD-post DEG (number of genes is shown in brackets). (**E**) Heat map showing cluster analysis of DEG contained in the GO term *skeletal muscle cell differentiation* enriched in CON-post (red arrows show differentially expressed genes between CON-post vs. HFD-post). (**F** and **G**) Top 20 TFBS enriched in CON-post and HFD-post DEG. (**H**) Transcript levels of TFs associated with the top 20 TFBS in CON and HFD mice. (**I**) Representative TFBS logos found in CON and HFD mice. n = 4 muscles per group.

The effect of acute maximal exercise on the skeletal muscle transcriptome was assessed following a single bout of treadmill running. Three hours post-exercise (post), 292 and 208 genes were up-regulated in quadriceps of CON-post and HFD-post mice, respectively (Supplementary Table S1). Moreover, 109 and 115 genes were reduced in CON-post and HFD-post mice, respectively (Supplementary Table S1). Approximately 60% of the up- and down-regulated genes detected in CON-post were not differentially expressed in HFD-post (Supplementary Fig. S2A), of which nearly 9% were already differentially expressed under basal conditions and, thus, reflect the robust effect of HFD on exercise-mediated skeletal muscle remodelling at the transcriptional level. While CON-post DEG were enriched for GO terms related with *muscle differentiation* and gene transcription (Fig. 3A), *cellular response to insulin stimulus* was the main biological proses enriched in HFD-post skeletal muscle (Fig. 3B). CON-post DEG had an enrichment of pathways that regulate the adaptive response to exercise in skeletal muscle, including AMPK and PPAR pathways (Fig. 3C and Supplementary Fig. S3A). Conversely, pathway analysis of HFD-post DEG shows remodelling of extracellular matrix genes and phosphatidylinositol 3-kinase (PI3K)-Akt signal activation (Fig. 3D and Supplementary Fig. S3B). Cluster analysis of the genes comprised in the GO term *skeletal muscle cell differentiation* demonstrated a generalised up-regulation in the post-exercise state, with few genes showing a milder response in HFD-post (Fig. 3E). HFD-post was associated with several DEG comprised in the GO term *cellular response to insulin stimulus*, whereas cluster analysis identified both up- and down-regulated genes (Supplementary Fig. S2B).

TFBS analysis showed a collection of 95 and 85 enriched TFBS in CON-post and HFD-post DEG, respectively (p < 0.01; Supplementary Table S2). Cluster analysis of the top 20 TFs associated with these motifs (Fig. 3F and G) did not show a gene expression pattern, though Kruppel-like factor 5 (Klf5) and early growth response 1 (Egr1) were induced by exercise, mainly in CON-post (Fig. 3H). Similar to pre-exercise TFBS analysis, we also found redundant motifs rich in C and/or G among the most significant TFBS, including the exercise-sensitive TFs Klf5 and Egr1 that indeed exhibit a highly similar motif (Fig. 3I). Nonetheless, we also identified TFBS with distinct motifs such as NFKB1 and E2F1 (Fig. 3F-I). TFBS analysis on post-exercise samples also predicted TFs that regulate energy metabolism both in muscle and non-muscle tissue such as E2F1 and KLF5, respectively^20,21^.

**Figure 4:**
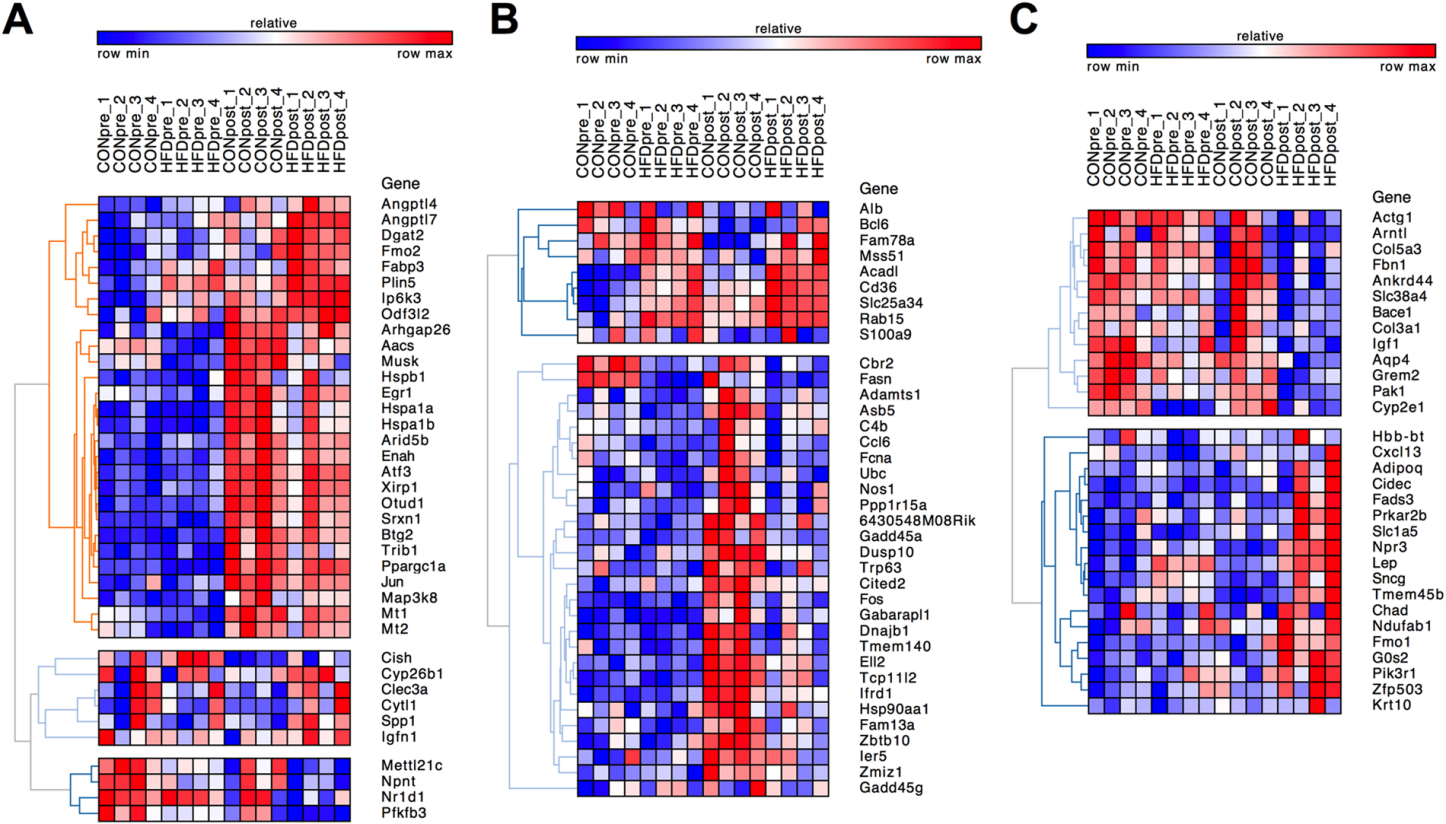
Identification of subset of genes linked with the detrimental effect of HFD on exercise-mediated skeletal muscle transcriptome remodelling. (**A**–**C**) Heat maps showing clusters of exercise-sensitive DEG in CON-post vs. HFD-post, including genes that are either (**A**) commonly regulated, (**B**) CON-post specific or (**C**) HFD-post specific. n = 4 muscles per group.

We also sought to detect down- or up-regulated genes in HFD-pre skeletal muscle of which exercise reverted their expression patterns (Supplementary Fig. S2C). Such categorisation showed genes with known function in skeletal muscle metabolism that we validated via quantitative PCR (qPCR) such as PPAR γ coactivator 1 α (PGC-1α) and NR4A3 (Supplementary Fig. S2D)^22,23^, whilst it allowed us to identify a number of novel genes with potential function in the development and treatment of metabolic diseases (Supplementary Fig. S2C). To pinpoint the subset of genes associated with the response to exercise in HFD mice we identified exercise-sensitive genes differentially expressed in CON-post vs. HFD-post. We detected 234 DEG in the post-exercise state (CON-post vs. HFD-post) of which only 16% were regulated by acute maximal exercise in both groups (Fig. 4A and Supplementary Fig. S2E). Accordingly, we observed subset of DEG specifically modulated in CON-post (17%; Fig. 4B and Supplementary Fig. S2E) and HFD-post (13%; Fig. 4C and Supplementary Fig. S2E). Clustering of these group-specific DEG showed different gene expression patterns, including genes with attenuated, enhanced or decreased expression in response to acute exercise and genes exclusively regulated by exercise in HFD-post mice (Fig. 4A–C). Collectively, this analysis uncovered several genes associated with the effects of HFD on exercise-mediated skeletal muscle remodelling.

### HFD enhances lipid accumulation and catabolism in skeletal muscle tissue

**Figure 5:**
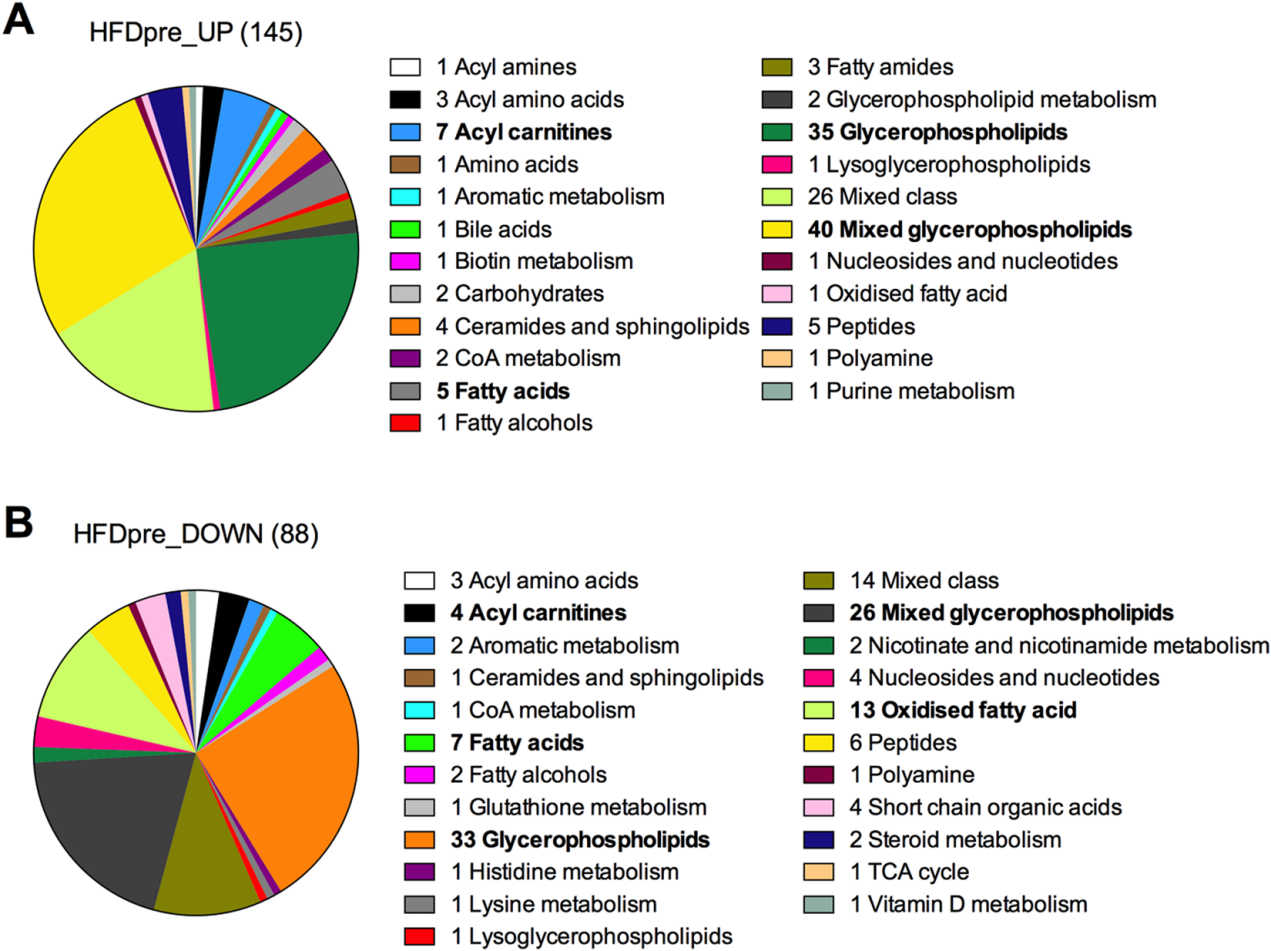
Effects of HFD on the skeletal muscle metabolome. (**A**) Increased and (**B**) decreased metabolites found in CON-pre and HFD-pre skeletal muscles classified according to their biological function. n = 6 muscles per group.

To complement transcriptome analysis, we conducted unbiased assessment of the skeletal muscle metabolome via untargeted metabolomics. HFD-pre skeletal muscles had higher and lower content of 145 and 88 analytes, respectively, compared to CON-pre (Supplementary Table S3). These analytes were associated both with single and multiple metabolites, whereas following classification by metabolic processes we found a distinct change in the content of metabolites related with lipid metabolism. Of the 145 up-regulated metabolites 52%, 5% and 3% were linked to glycerophospholipids, acyl-carnitines and fatty acids, respectively (Fig. 5A). Acetyl-CoA and tetradecenoyl-CoA were 2 and 2.7 fold higher in HFD-pre, respectively, potentially due to higher preference for lipid metabolism since HFD-pre also exhibited lower levels of S-Acetyldihydrolipoamide, suggesting lower pyruvate dehydrogenase activity. HFD significantly increased skeletal muscle ceramides among which sphingosine-1-phosphate (1.5 fold higher in HFD-pre) has been linked with the regulation of insulin sensitivity^24^. Similarly, though to a lower magnitude, down-regulated metabolites in HFD-pre also had a strong link with lipid metabolism, with glycerophospholipids representing 45% (Fig. 5B). Acute maximal exercise did not modify the skeletal muscle metabolome of CON-post mice 3 h post-exercise, whereas it exerted a mild effect in HFD-post skeletal muscle, with 4 and 10 up- and down-regulated analytes in HFD-post skeletal muscle, respectively (Supplementary Table S3). Although most of these analytes were associated with multiple metabolites, we found a significant reduction (−1.6 fold) of skeletal muscle triglycerides in HFD-post, suggesting lipid catabolism as an important biological process modulated by the interplay between diet-induce obesity and exercise.

### WACNA characterisation of integrated transcript-metabolite networks

**Figure 6:**
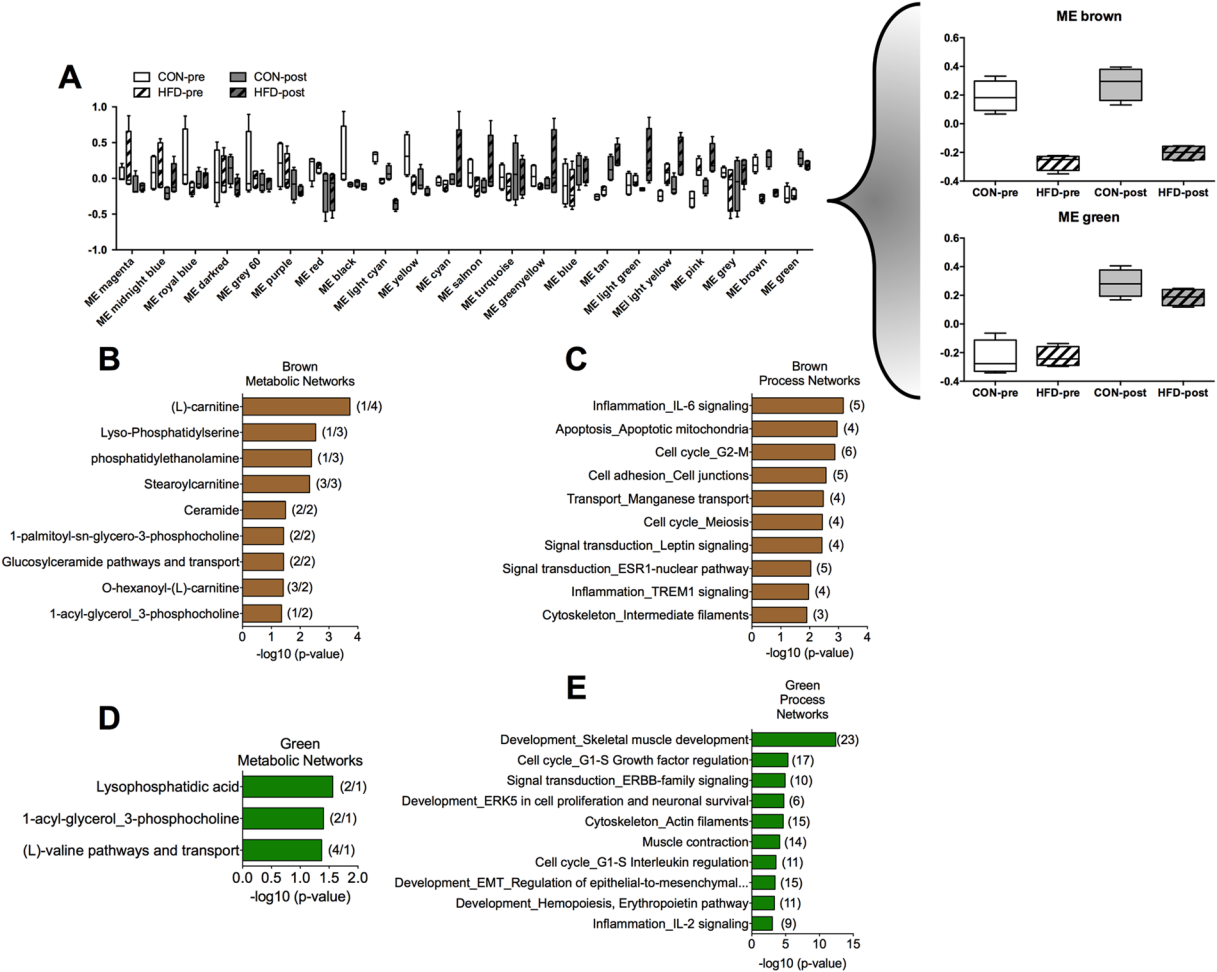
Integration of transcriptome and metabolome responses to HFD and acute maximal exercise. (**A**) Modules of correlated transcript and metabolites identified by WACNA analysis in CON and HFD skeletal muscles. (**B**–**E**) Pathway analysis was performed in subset of genes and metabolites comprised in the (**B** and **C**) brown and (**D** and **E**) green modules (number of metabolites/genes is shown in brackets). n = 6 muscles per group.

To define the association between changes in the skeletal muscle transcriptome and metabolome we performed WACNA. WACNA identified 22 modules of correlated genes and metabolites with distinction between modules influenced by HFD and exercise (Fig. 6A). Of particular interest, the brown module was highly regulated by diet, while the green module was regulated by exercise both in CON and HFD mice (Fig. 6A, right panel). Pathway analysis of correlated genes and metabolites in the brown module demonstrated a strong association with lipid metabolism, characterized by pathways controlling glycerophospholipid and acyl carnitine metabolism (Fig. 6B; Supplementary Table S4). Independent analysis of brown module transcripts showed enrichment of terms related with inflammation and cell cycle (Fig. 6C; Supplementary Table S4). Consistent with the lack of effects of acute exercise on the skeletal muscle metabolome 3 h post-exercise, combined analysis of transcript and metabolites in the green module showed minor enrichment of metabolic networks (Fig. 6D; Supplementary Table S4). However, the green module showed a strong enrichment of genes associated with skeletal muscle development and function (Fig. 6E; Supplementary Table S4). Therefore, these data further demonstrate the robust effect of HFD-induced obesity on the skeletal muscle metabolome, with acute maximal exercise having a major function in skeletal muscle transcriptome remodelling and a minor effect on metabolism when comparing CON and HFD 3 h post-exercise.

## Discussion

Skeletal muscle insulin resistance and metabolic dysfunction are central pathogenic factors in the development of metabolic disorders, such as T2D^25–30^. Gene transcription is a key process controlling skeletal muscle phenotype and, consequently, metabolic health. Both pathological and physiological cues coupled with impaired and improved insulin sensitivity, respectively, extensively remodel the skeletal muscle transcriptome^7,13,14,31^. In the present study we used unbiased approaches to assess the effect of exercise and diet on the skeletal muscle transcriptome (RNA-seq) and metabolome (untargeted metabolomics), in addition to studying the interaction between both responses (using WACNA). In doing so, we report that under basal conditions, HFD enhanced skeletal muscle lipid catabolism at the transcriptional level, while promoting the accumulation of glycerophospholipid and acyl carnitine species at the metabolic level. We also demonstrate that HFD is linked to a different transcriptional response to acute maximal exercise compared to CON mice, which in HFD mice comprised mainly GO terms associated with the regulation of skeletal muscle plasticity and gene transcription. Finally, we successfully implemented WACNA for transcript-metabolite correlation analysis, which further demonstrated the predominant effect of HFD and exercise on lipid metabolism and gene transcription, respectively.

Concurrent with the development of glucose intolerance, our data demonstrates that HFD result in a robust increased in skeletal muscle lipid catabolism at the transcriptional level. Interestingly, short- and long-term feeding of a HFD with different composition (45% kcal from palm oil) can induce similar effects on lipid metabolism in mouse skeletal muscle^32^. Moreover, human skeletal muscle from pre-diabetic subjects has also been found to have higher expression of genes controlling fatty acid catabolism^31^. Thus, the coordinated up-regulation of genes controlling lipid catabolism is a common response to lipid overload, probably as a compensatory mechanism. Our data further imply the nuclear receptors PPARs as central regulators of such transcriptional response, which is supported by both pathway and TFBS analysis. Interestingly, PPARα overexpression in skeletal muscle concurrently increases fatty acid oxidation and impairs insulin sensitivity^33^. In contrast, overexpression of a hyperactive form of PPARδ in skeletal muscle results in positive effects on glucose tolerance^34^, suggesting that selective PPARs isoform activation might take place following different environmental cues. Moreover, consistent with this transcriptional response, we found a significant increase in acetyl-CoA and acyl carnitine species in skeletal muscle from HFD-pre mice. At the whole body level, obesity in humans and HFD in rodents also increase lipid oxidation as reflected by lower respiratory exchange ratio^30,35,36^. These data suggest enhanced lipid catabolism, supporting abnormal energy substrate partitioning as an important factor contributing to the development of skeletal muscle insulin resistance^25^. Despite lower malic acid levels, we did not find major changes in TCA cycle and OXPHOS related genes or metabolites in HFD-pre skeletal muscle. We further validated the effect of HFD on lipid metabolism via WACNA analysis. The main module reflecting correlated genes and metabolites linked to the diet effects (brown module) comprised genes with known function in muscle biology, lipid catabolism and glycerophospholipid synthesis such as MEF2A, monoglyceride lipase and phosphatidylserine decarboxylase, respectively. This module also comprised glycerophospholipids (e.g. phosphatidylserines) and acyl carnitine species (e.g. stearoyl carnitine), further suggesting abnormal lipid metabolism as a key biological process involved in the pathogenesis of insulin resistance. However, it remains to be investigated whether acute exercise exerts transient changes in the skeletal muscle metabolome and whether it correlates with the transcriptional response, which could probably be detected at an earlier post-exercise time point^37^.

Exercise training is well known to remodel the skeletal muscle transcriptome and to improve insulin sensitivity, though the early transcriptional responses leading to such adaptations remain poorly understood. In the long-term, exercise co-ordinately enhances the expression of genes controlling energy metabolism, whereas acute exercise seems to induce different transcriptional networks^13^. We found gene transcription as the main biological process induced by acute maximal exercise in mice fed CON diet when measured 3 h post-exercise. In fact, although the GO term *skeletal muscle cell differentiation* was highly significant, different TFs and transcriptional co-regulators primarily composed this GO term. TFBS analysis revealed a subset of exercise-sensitive TFs motifs enriched in CON and HFD mice, suggesting that their regulation is completely or partially independent of nutritional factors. Among these genes, only Egr1 and Klf5 expression and motif enrichment were enhanced by exercise, though obesity had a blunting effect. Interestingly, Egr1 has been found to induce SIRT1 expression^38^, while Klf5 can modulates PPARδ activity^20^, thus both TFs represent potential mediators of exercise-induced skeletal muscle plasticity. Overall, HFD completely blunted the enrichment of GO terms linked to gene transcription, further exposing the effects of HFD on exercise-mediated adaptations. Similar effects on PGC-1α and a few of its target genes have been observed in skeletal muscle from obese subjects, leading to the idea that HFD and insulin resistance impair the effects of exercise on skeletal muscle remodelling at the transcriptional level^39,40^. Accordingly, here we have demonstrated that this phenomenon can also take place in mouse skeletal muscle at a large scale (transcriptome), affecting several transcriptional networks.

Both GO and pathway analysis revealed insulin signalling as a central biological process induced by exercise in HFD mice. When comparing transcriptional responses in CON-post and HFD-post, we identified genes with both known and unknown function in skeletal muscle metabolism. For example, HFD abrogated the induction of genes linked to energy metabolism in non-muscle tissues, such as Cbp/p300-interacting transactivator, with Glu/Asp-rich carboxy-terminal domain 2 (Cited2)^41^, dual specificity phosphatase 10 (Dusp10)^42^ and transformation related protein 63 (Trp63)^43^. Genes with therapeutic potential of which exercise reverted the abnormal expression patterns included genes with known function in skeletal muscle metabolism and exercise-mediated adaptations such as NR4A3^23^ and PGC-1α^22^. Interestingly, Fu *et al*. have recently found that 8 weeks of HFD also impairs the effects of exercise training (6 weeks) on mouse skeletal muscle transcriptome^44^. Unlike acute exercise, chronic exercise appears to regulate different transcriptional programs both in CON and HFD fed animals, including genes linked to energy metabolism and mitochondrial function^44^. WACNA analysis revealed several correlated genes linked to development, cell cycle and muscle contraction in the main exercise-sensitive module (green module), whereas energy metabolism-related terms were not enriched. Interestingly, the green module comprised several genes controlling gene transcription, including histone deacetylase 5, Jun, MEF2C and myelocytomatosis oncogene. This data also suggests gene transcription as a key biological process induced by acute exercise and, potentially, mediating long-term skeletal muscle adaptations, whereas HFD seem to disrupt this exercise-sensitive transcriptional network.

Overall, we have identified skeletal muscle lipid metabolism and gene transcription as central biological processes linked with glucose intolerance and exercise-mediated adaptations, respectively. The unbiased approaches utilised in this study allowed us to comprehensively characterise the skeletal muscle transcriptome and metabolome, which revealed a broad range of novel metabolite (primarily under basal conditions) and transcript targets. Implementing WACNA to these datasets provided an innovative approach to integrate unbiased transcriptome and metabolome datasets. Besides supporting independent RNA-seq and metabolomics analysis, WACNA identified related transcript and metabolite networks sensitive to exercise and HFD. Therefore, we anticipate that the independent and integrated datasets reported herein will provide an important resource for studies focused on skeletal muscle physiology in the context of metabolic diseases.

## Methods

### Animals

We bought sixteen-week-old C57BL/6NTac male mice (Taconic Biosciences), which had been housed under standard conditions with free access to water and either control diet (CON; 10% kcal from fat, 70% kcal from carbohydrate, 20% kcal from protein) or high-fat diet (HFD; 60% kcal from fat, 20% kcal from carbohydrate, 20% kcal from protein; Research Diets, #D12492) for 10 weeks. All methods and experiments were carried out in accordance with relevant guidelines and regulations, and with the approval of the Animal Care Program at the University of California, San Diego.

### Body composition

Lean and fat mass was measured by magnetic resonance (EchoMRI^TM^).

### Oral glucose tolerance test (OGTT)

Mice were fasted for 4 h and, subsequently, 2 g/kg body weight of dextrose was delivered via oral gavage. Blood glucose was measured with a standard meter, with samples obtained from the tail vein before or 10, 20, 30, 45, 60, 90 and 120 min after the dextrose gavage.

### Maximal exercise test

One day before the test, fed (ad libitum) mice were acclimatized to the treadmill (Columbus Instruments) by performing one session of 10 min at 8 m/min, 5 min at 0 m/min and 10 min at 10 m/min with 8.5° incline. The next morning mice were fasted for 3 h, following which they were placed in the treadmill (8.5° incline) for 5 min at 0 m/min, with the test starting at 8 m/min for 3 min and the speed increased 2 m/min every 3 min until exhaustion. Subsequently, mice were placed in their cages with free access to water but no food. A group of CON and HFD mice under basal conditions was used as pre-exercise control (pre). Importantly, CON-pre and HFD-pre groups underwent the same treadmill acclimatization and fasting period as the exercise groups. Muscles samples were collected from anesthetized (2.5% isoflurane) mice under basal conditions or 3 h after maximal exercise (post).

### RNA isolation and quantitative PCR (qPCR)

Total RNA extraction and reverse transcription was performed using RNeasy Mini Kit (QIAGEN, #74104) and iScript™ cDNA Synthesis Kit (Bio-Rad Laboratories, #170-8891), respectively. Relative mRNA was quantified by qPCR on a Mastercycler^®^ ep realplex (Eppendorf) using SsoAdvanced™ Universal SYBR^®^ Green Supermix (Bio-Rad, #172-5270). The ΔΔC_T_ method was used for analysis, with TATA binding protein (TBP) as endogenous control.

### RNA sequencing (RNA-seq)

Libraries were prepared with TruSeq Stranded mRNA Library Prep Kit (Illumina). Sequencing was performed at the Institute for Genomic Medicine of the University of California using the HiSeq. 2500 (Illumina), which resulted in ~20 M reads per sample. Reads were trimmed to remove adapter sequences and quality checked using fastqc software (http://www.bioinformatics.babraham.ac.uk/projects/fastqc/). Reads were aligned using TopHat to mm9 version of the mouse genome^45^. The expression level of genes and comparison between different conditions to find significant changes in expression was done using cufflinks^46^. Gene Ontology and pathway analysis was performed with DAVID 6.8 Beta^47,48^ and KEGG (Kyoto Encyclopedia of Genes and Genomes) database (http://www.kegg.jp/kegg/pathway.html)^49–51^, respectively, while TFBS analysis was performed with Pscan using JASPAR 2016 database and a promoter region of −950 to +50 bp^52^. Heat maps and hierarchical clustering was performed with GENE-E (http://www.broadinstitute.org/cancer/software/GENE-E/index.html).

### Untargeted metabolomic analysis

#### Sample preparation

50 mg of powdered muscle tissue was mixed with 1000 µL of methanol:water:chloroform (2.5:1:1 [v/v/v]) and homogenised in a Precellys24 (Bertin Technologies, Stretton Scientific U.K.) at 6800 Hz for 2 × 30 s cycle followed by shaking for 10 min. Samples were centrifuged (10,000 g, 3 °C, 5 min) followed by transfer of 1000 µL of the supernatant to a clean 2 mL microcentrifuge tube. 500 µL of HPLC grade water was added followed by vortex mixing and centrifugation (10,000 g, 3 °C, 5 min) to induce phase separation. 600 μL of the upper polar phase (methanol/water) and 100 μL of the lower non-polar phase (chloroform) were transferred in to separate clean 2 mL centrifuge tubes and dried by centrifugal vacuum evaporation for 6 h. Samples were stored at −80 °C until analysis.

#### UHPLC-MS

All samples were analysed applying an UltiMate U3000 RSLC UHPLC system coupled to an electrospray Q-Exactive mass spectrometer. The polar phase samples were analysed applying HILIC-MS after being reconstituted in 100 μL of solvent (95/5 acetonitrile/water) and the non-polar phase samples were analysed applying reversed phase C_18_-MS after being reconstituted in 100 μL of solvent (50/50 water/methanol). After reconstitution, 20 µL of each samples was pooled in to a QC sample to quantify technical reproducibility.

#### HILIC-MS

A Accucore 150-Amide HILIC UHPLC column (100 mm × 2.1 mm 2.6 µm, Thermo-Fisher Ltd., UK) was applied. A gradient elution applied two mobile phases (A- 95% acetonitrile in water +5 mM Ammonium Formate at pH 3; B- water +5 mM Ammonium Formate at pH 3) as follows: start (95%A) and operates as follows: 0 to 1 min. A is constant at 95%; from 1 to 12 min. B increases from 5 to 45%; from 12 to 15 min. B is kept constant at 45%; from 15 to 16 min. A increases from 55% to 95%; from 16 to 21 min. 5 µL of sample was injected and the mass spectrometer conditions were spray voltage (3.5 kV (ESI−) and +4.5 kV (ESI+)), sheath gas 40; aux gas 15; sweep gas 0, S lens 100; Resolution 35,000 (FWHM, m/z 200), capillary temperature 300 °C; ESI heater temperature 300 °C. All samples were analysed applying positive-negative ion mode switching with data collected in the m/z range 100–1000. All samples were stored in the autosampler at 4 °C. Ten QC samples were analysed at the start of the analysis followed by a QC sample after every 6^th^ biological sample and two QC samples at the end of the analytical run. Biological samples were randomised across the analytical batch.

Solvent A is constituted by acetonitrile 95% + Ammonium Formate 5 mM at pH 3 and solvent B is constituted by H_2_O+ Ammonium Formate 5 mM at pH 3. The gradient elution conditions are: from 0 to 1 min. A is constant at 95%; from 1 to 12 min. B increases from 5 to 45%; from 12 to 15 min. B is kept constant at 45%; from 15 to 16 min. A increases from 55% to 95%; from 16 to 21 min. A is constant at 95%.

Reversed phase C_18_-MS: A Hypersil Gold UHPLC C_18_ column (100 mm × 2.1 mm 1.9 µm, Thermo-Fisher Ltd.) was applied. A gradient elution applied two mobile phases (A-99.9/0.1 water/formic acid; B-99.9/0.1 methanol/formic acid) as follows: start (95%A) for 2 minutes, linear ramp to 95%B over 7 min, hold for 3 min, step return to 95%A and hold for 3 min. 5 µL of sample was injected and the mass spectrometer conditions were spray voltage (−3.5 kV (ESI−) and +4.5 kV (ESI+)), sheath gas 40; aux gas 15; sweep gas 0, S lens 100; resolution 35,000 (FWHM, m/z 200), capillary temperature 300 °C; ESI heater temperature 300 °C. All samples were analysed applying positive-negative ion mode switching with data collected in the m/z range 100–1000. All samples were stored at 4 °C. Ten QC samples were analysed at the start of the analysis followed by a QC sample after every 6^th^ biological sample and two QC samples at the end of the analytical run. Biological samples were randomised across the analytical batch.

#### Data processing and statistical analysis

Raw data (.RAW) were converted to mzML files applying ProteoWizard followed by data processing applying XCMS software as described previously^53^. Data were exported as a data matrix of metabolite feature (m/z-retention time pair) vs. sample with associated chromatographic peak areas for a detected metabolite. Each metabolite feature with a relative standard deviation calculated for QC samples greater than 20% and not detected in greater than 70% of samples were removed prior to univariate and multivariate data analysis as described previously^54^. All metabolite features were annotated according to level 2 of the MSI reporting standards^55^ applying PUTMEDID_LCMS, as previously described^56^. The processed data were analysed in R applying the unsupervised multivariate principal components analysis (PCA) and the univariate non-parametric Wilcoxon–Mann–Whitney test. Correction for false discovery rate was applied using the Benjamini–Hochberg procedure. The fold change was calculated including 95% confidence limits. Metabolites were manually clustered in to classes defining similar chemical structure or metabolic pathway to identify biologically relevant and robust metabolic changes.

### Weighted Analyte Correlation Network Analysis (WACNA)

To perform an integrated analysis of the transcriptomic and metabolomic data, we generalised Weighted Gene Co-expression Network Analysis. First, missing values in the metabolomics data were imputed using k-nearest neighbours imputation with k = 3. Second, to reduce the dimensionality of the final matrix only the top 20% of data (by variance) for each dataset (metabolomics from positive ion spectroscopy, from negative ion spectroscopy and RNA-Seq) were carried forwards. These data matrices were combined, mean centred and z-scaled. Finally, WGCNA was carried out as described elsewhere^15^. Modules of correlated analytes (metabolites and transcripts) were identified using a measure of topological overlap and each named with a colour for easy reference. For each module, a module eigengene was computed (defined as the first principal component). This eigengene provided an aggregate measure for all the analytes in the module and was used to identify modules of interest (i.e. those that were affected by experimental interventions). Finally, seven modules of interest identified thus were passed into MetaCore (Thompson Reuters) for enrichment analysis and pathway mapping. All analyses prior to MetaCore were conducted in R; code is available on request.

### Statistical analysis

Values are expressed as mean ± SEM. Statistical significance was determined with unpaired two-tailed t-tests or two-way ANOVA with Tukey post hoc test. Significance was considered with a *p* < 0.05.

### Data availability

The RNA-seq data is available at Gene Expression Omnibus (accession number: GSE97718).

## Electronic supplementary material

Supplementary information

Table S1

Table S2

Table S3

Table S4
