# Supplementary material for: Exercise and high-fat feeding remodel transcript-metabolite interactive networks in mouse skeletal muscle
Source: Sci Rep. 2017 Oct 18;7:13485. doi: 10.1038/s41598-017-14081-w (PMC5647435; doi:10.1038/s41598-017-14081-w)
Supplement: Supplementary file 1 — Supplementary information [file 41598_2017_14081_MOESM1_ESM.pdf]

Supplementary information

Exercise and high-fat feeding remodel transcript-metabolite interactive networks in mouse skeletal muscle

Joaquin Perez-Schindler[1,2,8,*], Aditi Kanhere[3,§], Lindsay Edwards[4,§], J. William Allwood[3,5,9], Warwick B. Dunn[3,5], Simon Schenk[6,7] & Andrew Philp[1,2,*]

[1]MRC-ARUK Centre for Musculoskeletal Ageing Research, [2]School of Sport, Exercise and Rehabilitation Sciences, University of Birmingham, Birmingham, B152TT, UK. [3]School of Biosciences, University of Birmingham, Birmingham, B152TT, UK. [4]Respiratory Therapy Area Unit, GlaxoSmithKline Medicines Research Centre, Stevenage, SG1 2NY, UK. [5]Phenome Centre Birmingham, University of Birmingham, Birmingham, B152TT, UK. [6]Department of Orthopaedic Surgery, University of California San Diego, La Jolla, CA 92093-0863, USA. [7]Biomedical Sciences Graduate Program, University of California San Diego, La Jolla, CA 92093-0863, USA.

[8]Current address: Biozentrum, University of Basel, Basel, Switzerland.

[9]Current address: Environmental and Biochemical Sciences, The James Hutton Institute, Dundee, Scotland.

[§] Equal contribution.

*Correspondence and requests for materials should be addressed to J.P.S (j.perez-schindler@bham.ac.uk) and A.P (a.philp@bham.ac.uk).

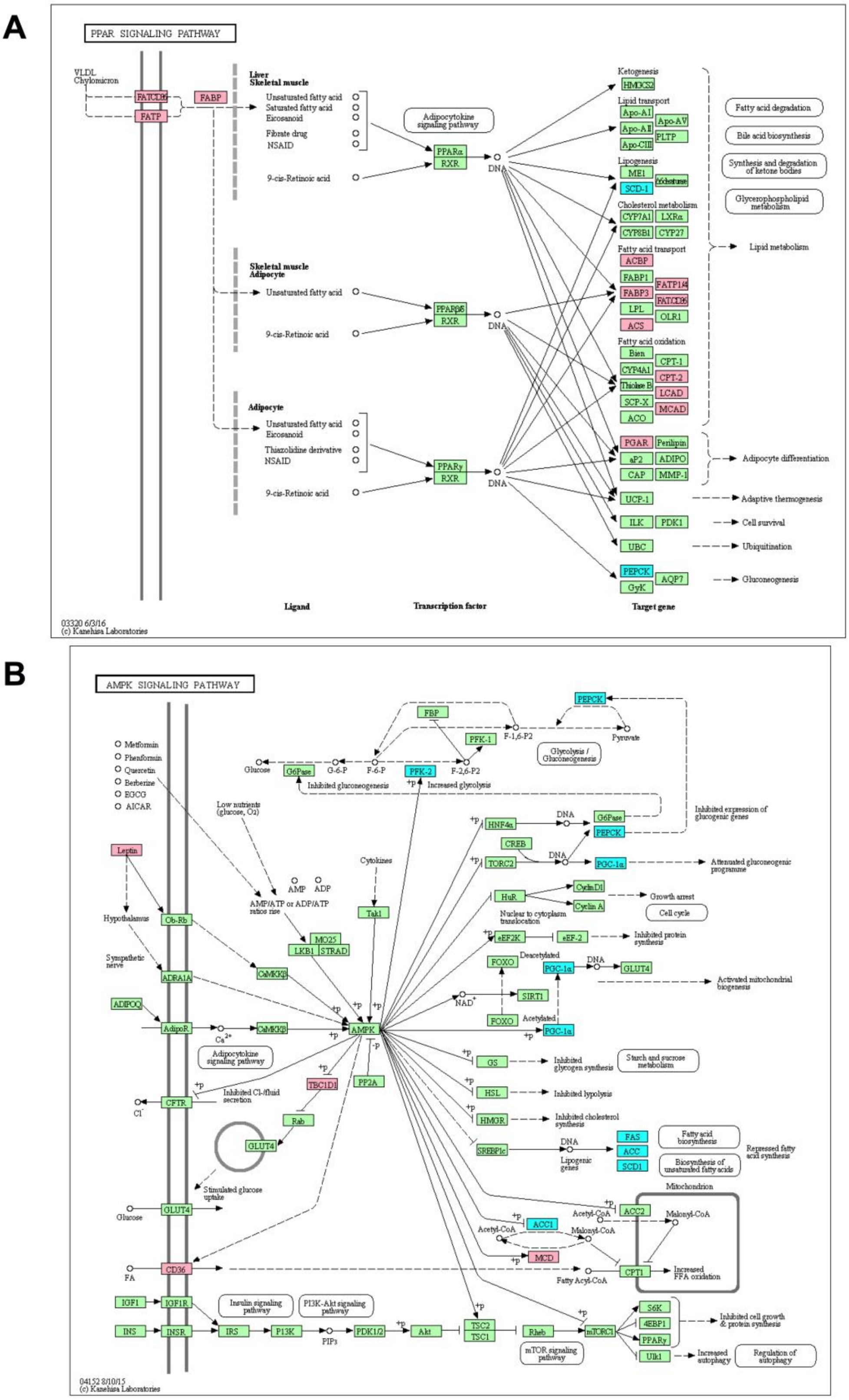

Figure S1. KEGG pathway analysis of HFD-pre DEG. (A) PPAR and (B) AMPK signalling pathways showing up- (pink) and down-regulated (cyan) genes in HFD-pre DEG. n= 4 muscles per group. PPAR signaling pathway (map03320) and AMPK signaling pathway (map04152) maps were obtained with permission from KEGG database (http://www.kegg.jp/kegg/pathway.html)[1-3].

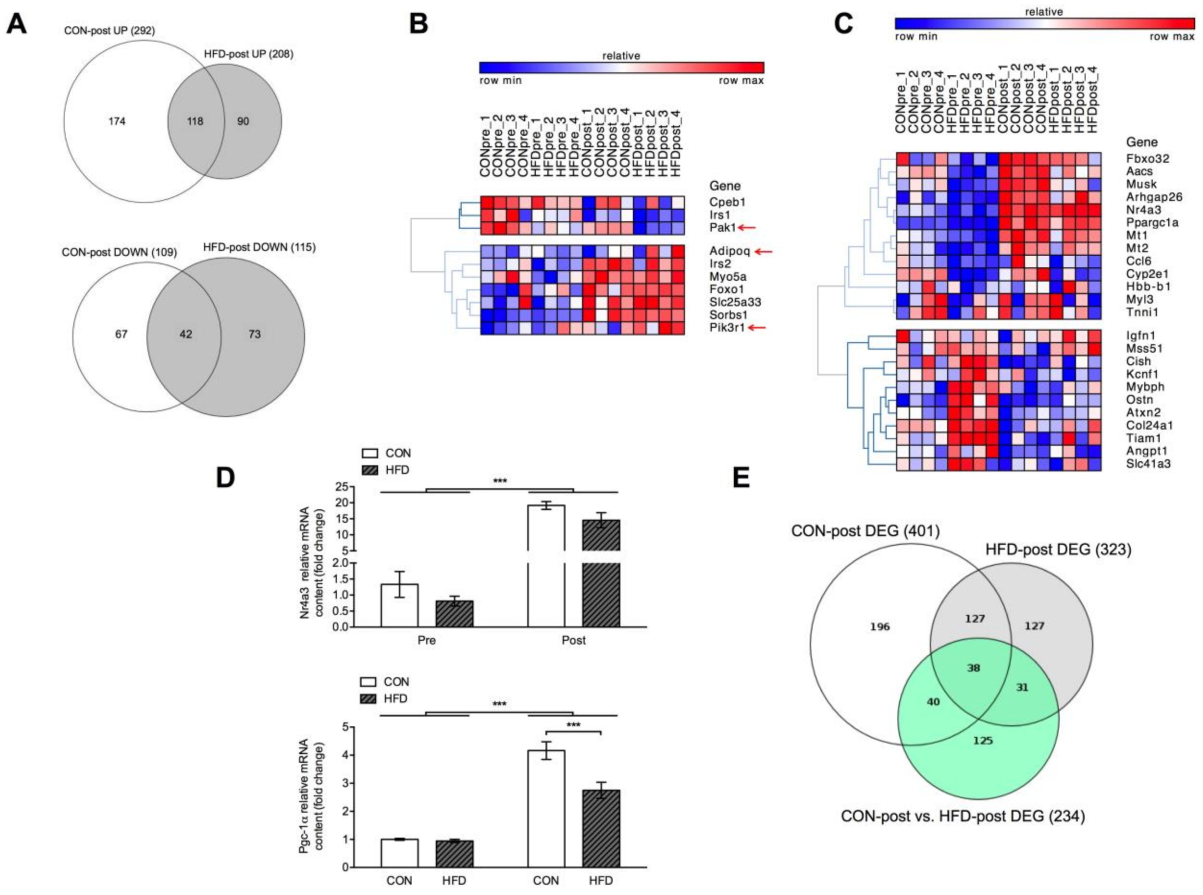

Figure S2. Transcriptional response to exercise in CON and HFD mice. (A) DEG in CON-post and HFD-post skeletal muscles (number of genes is shown in brackets or circle regions). (B) Heat map and clustering of genes comprised in the GO term " cellular response to insulin stimulus" (red arrows show differentially expressed between CON-post vs. HFD-post). (C) Subset of genes deregulated by HFD under basal conditions of which acute maximal exercise normalized their expression levels. (D) qPCR analysis of Nr4a3 and Pgc-1α mRNA levels (n= 6 muscles per group). (E) Venn diagram with subsets of exercise-sensitive genes of which HFD affected the response to exercise.

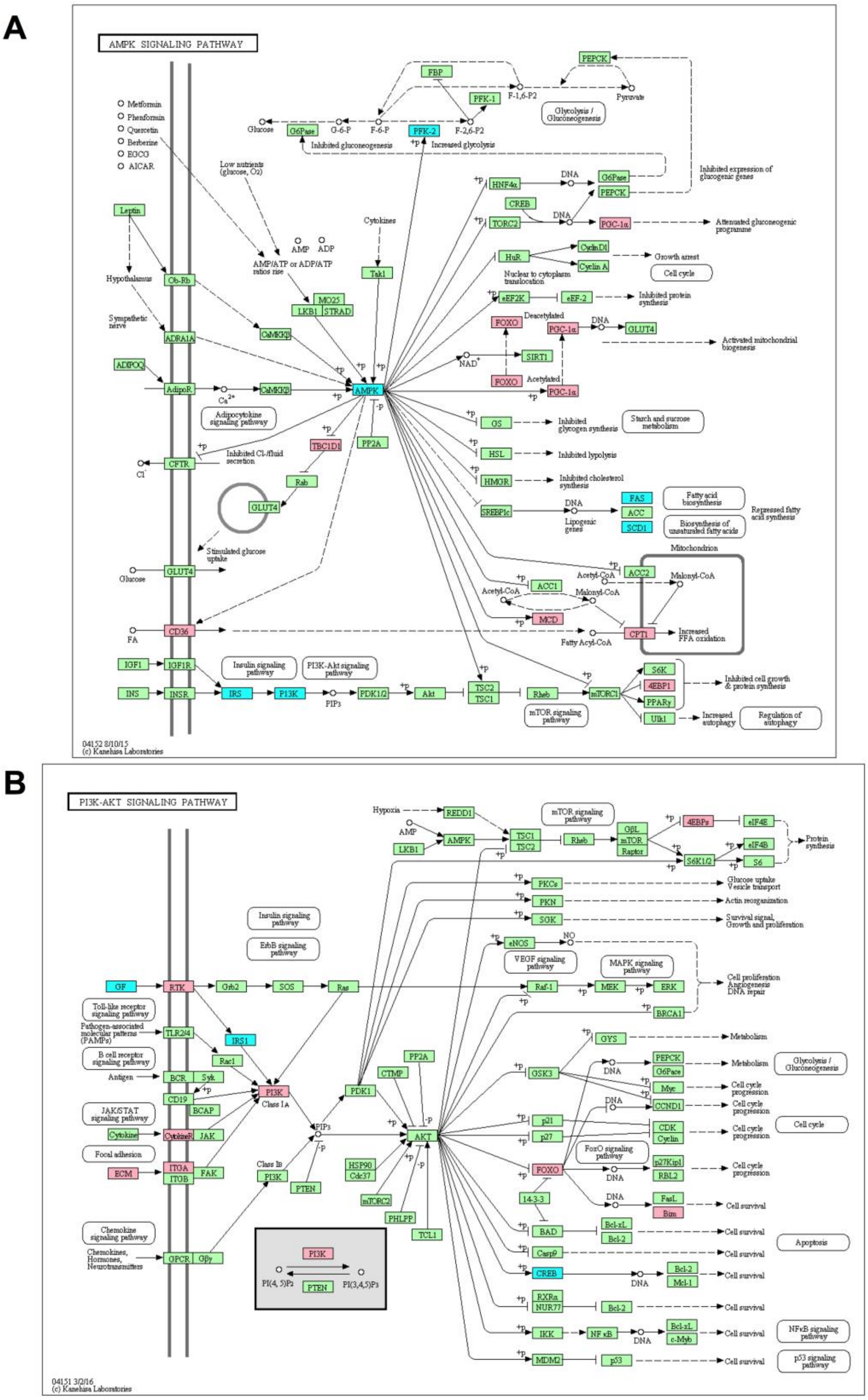

Figure S3. KEGG pathway analysis of CON-post and HFD-post DEG. (A) AMPK and (B) PI3K-Akt signalling pathways showing up- (pink) and down-regulated (cyan) genes in CON-post and HFD-post DEG. n= 4 muscles per group. AMPK signaling pathway (map04152) and PI3K-Akt signaling pathway (map04151) maps were obtained with permission from KEGG database (http://www.kegg.jp/kegg/pathway.html)[1-3].

Supplementary References

1 Kanehisa, M., Furumichi, M., Tanabe, M., Sato, Y. & Morishima, K. KEGG: new perspectives on genomes, pathways, diseases and drugs. *Nucleic acids research* **45**, D353-D361, doi:10.1093/nar/gkw1092 (2017).

2 Kanehisa, M., Sato, Y., Kawashima, M., Furumichi, M. & Tanabe, M. KEGG as a reference resource for gene and protein annotation. *Nucleic acids research* **44**, D457-462, doi:10.1093/nar/gkv1070 (2016).

3 Kanehisa, M. & Goto, S. KEGG: kyoto encyclopedia of genes and genomes. *Nucleic acids research* **28**, 27-30 (2000).
